# Experimental Demonstration of Underwater Acoustic Scattering Cancellation

**DOI:** 10.1038/srep13175

**Published:** 2015-08-18

**Authors:** Charles A. Rohde, Theodore P. Martin, Matthew D. Guild, Christopher N. Layman, Christina J. Naify, Michael Nicholas, Abel L. Thangawng, David C. Calvo, Gregory J. Orris

**Affiliations:** 1 National Research Council Research Associate Program, U.S. Naval Research Laboratory, Code 7160, Washington, DC 20375, USA; 2 U.S. Naval Research Laboratory, Code 7160, 4555 Overlook Ave., Washington, DC 20375, USA

## Abstract

We explore an acoustic scattering cancellation shell for buoyant hollow cylinders submersed in a water background. A thin, low-shear, elastic coating is used to cancel the monopole scattering from an air-filled, neutrally buoyant steel shell for all frequencies where the wavelength is larger than the object diameter. By design, the uncoated shell also has an effective density close to the aqueous background, independently canceling its dipole scattering. Due to the significantly reduced monopole and dipole scattering, the compliant coating results in a hollow cylindrical inclusion that is simultaneously impedance and sound speed matched to the water background. We demonstrate the proposed cancellation method with a specific case, using an array of hollow steel cylinders coated with thin silicone rubber shells. These experimental results are matched to finite element modeling predictions, confirming the scattering reduction. Additional calculations explore the optimization of the silicone coating properties. Using this approach, it is found that scattering cross-sections can be reduced by 20 dB for all wavelengths up to *k*_0_*a* = 0.85.

Over the past decade, metamaterial based coordinate transformation coatings have been suggested as a path toward total scattering reduction in both electromagnetic^1^,^2^,^3^,^4^ and acoustic systems^5^,^6^,^7^. These coatings guide waves around an object while minimizing the scattering interaction with the object. However, transformation-based coatings have not been demonstrated for acoustic waves in open water because the requisite material properties are difficult to achieve, particularly for coatings which are thin on the scale of the scattering object^6^. An alternate method to achieve scattering reduction, originally studied for electromagnetic waves as plasmonic coatings^8^,^9^,^10^ and later extended to acoustics^11^, is to add a coating whose material properties result in the cancellation of the most significant scattered multipole modes over a finite frequency bandwidth. To date, this form of non-resonant acoustic scattering cancellation has primarily been studied theoretically and computationally for uniform objects, such as solid stainless steel spheres and cylinders^12^ with one recent exception examining hollow elastic spheres^13^. Experimental demonstrations of this effect are rare, and have only recently been conducted for electromagnetic waves^14^ in solid cylindrical systems. In acoustics, experimental demonstrations have been limited to objects in air for single frequency and single incident direction cancellation shells on solid cylinders and spheres^15^,^16^ and for hollow cylinders in the zero frequency limit using pentamode shells^17^. Our scattering cancellation results are unique in that we experimentally demonstrate an omnidirectional acoustic scattering reduction for hollow cylindrical shells in water. Additionally, our system is comprised of a simple, low-shear, isotropic elastomer coating which is not limited to coating rigid objects, and works in water for all wavelengths greater than three times the object diameter.

Acoustic scattering cancellation enables the elimination of the leading order scattering modes, mitigating the scattering in the entire region around an object in all directions. In previous work we have used multiple scattering theory to examine the long-wavelength effective acoustic properties of air-filled elastic cylinders and the conditions under which they become impedance matched to a water background^18^. However, we found that the shell thicknesses needed to meet the impedance matching condition for most common materials resulted in a density mismatch with the background medium. By contrast, in this study we start with the fixed condition that an uncoated elastic shell, with outer radius *a*, is density matched to the background aqueous medium through the scaling of its inner radius, *b*. The geometry of the coated shells and the experimental setup are illustrated in Fig. 1.

An object’s dipole scattering mode is reduced to zero in the quasi-static limit when its effective density is equal to the background medium density, and the major contributor to the scattering is from the object’s effective compressibility (the monopole mode)^18^. We therefore introduce an elastic coating with thickness, *T*, that will scale the effective bulk modulus of the scatterer, while minimally impacting its effective density. To be clear, throughout we will refer to the hollow steel cylinder as a shell, the combined steel shell and air core as a hollow buoyant cylinder (HBC) and the outer compliant cylindrical shell as a coating. For a given set of material properties, the correct coating and shell thicknesses will result in the simultaneous matching of both the impedance and sound speed to the background due to the elimination of the monopole and dipole scattering modes.

## Results

### Formulating a broadband cancellation coating

To find the limits of the parameter space in which our coating’s physical properties must exist, we consider the scattering properties of multilayer cylinders in a plane-wave field with wavevector magnitude *k*_0_ = 2*π*/*λ*. The monopole scattering mode of a multilayer elastic cylinder is, in general, a complicated function of its constituent component densities, bulk and shear moduli. When *T* ≪ *a*, the coating bulk modulus, *κ*_c_, needed for monopole cancellation can be expressed as^19^,^13^:


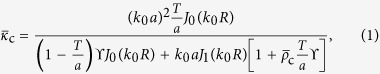


where the over-bar indicates normalization by the background medium: 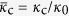, 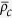 is the normalized coating density, *J*_*n*_ is the *n*^th^ order Bessel function, *R* = *a* + *T* is the total radius, and ϒ is a term which accounts for the finite impedance of the uncoated object^13^. Eq. (1) indicates the coating modulus needed to achieve monopole cancellation for any arbitrary (multilayer, anisotropic, etc.) cylindrical object at any single frequency. However, due to the non-resonant nature of the cancellation, this effect can lead to a broadband scattering reduction away from the design frequency^11^,^12^,^13^.

In the quasi-static limit (*k*_0_*a* ≪ 1) the zeroth-order (monopole) and first-order (dipole) scattering coefficients of a multilayer cylindrical scatterer can be separated into independent respective contributions from the object’s effective bulk modulus (*κ*_eff_) and effective density (*ρ*_eff_)^18^,^20^,^21^. Additionally, the expression for ϒ simplifies considerably^13^, and for a cylindrical object reduces to:


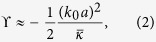


where the background normalized 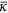 is the effective bulk modulus of the uncoated HBC. 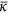 can be obtained from effective medium theory and can be found elsewhere^22^,^23^. For a soft rubber coating a thin metal shell, where the HBC can be approximated as an effective fluid, it follows that Eq. (1) can be replaced in the quasi-static limit by^19^:


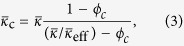


where *ϕ*_*c*_ = (*a*/*R*)^2^ is the shell to coating ratio. We note that monopole cancellation is achieved when 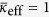
18. From Eq. (3) it is apparent that, in the long wavelength case, the bulk modulus of the coating layer needed for monopole scattering cancellation depends only on the relative thickness of the coating, and the bulk modulus of the uncoated HBC.

Also in the quasi-static limit, the normalized coating density, 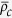, which results in non-resonant dipole cancellation, is given by the volume fraction expressions^19^:


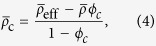


where, 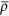 is the normalized effective density of the uncoated HBC. Similarly 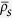 is the normalized effective density of the shell:
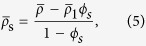

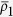
 is the normalized density of the inner air core, and *ϕ*_*s*_ = (*b*/*a*)^2^ is the core to shell size ratio. For a fixed shell material density *ρ*_s_, neutral buoyancy is set by the correct choice of shell thickness, *a*-*b*, given by Eq. (5). The sole dependence of the dipole scattering on the effective density as shown in^20^ ensures cancellation of the dipole scattering when 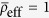. Thus, scaling the inner diameter to obtain a neutrally buoyant HBC results in the elimination of dipole scattering when 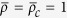.

With a shell thickness prescribed by Eq. (5) and a coating bulk modulus given by either Eq. (1) or (3), an air-filled, hollow scatterer can be constructed which simultaneously eliminates the scattered monopole and dipole modes. At low to moderate frequencies, this can lead to a significant reduction in the scattering strength of an object. For a submerged, neutrally buoyant shell this reduction can be observed in Fig. 2a as a function of the parameter space for the normalized coating’s physical properties at a fixed coating filling fraction, *ϕ*_*c*_ = 0.64 and *k*_0_*a* = 0.2. These values correspond to the *T* = 1 mm shell, coating an *a* = 4 mm HBC, used in our experiments at 10 kHz. Fig. 2a is a color map of the scattering cross-section ratio (*σ*_0_) of a coated HBC scatterer (*σ*_*C*_) to an uncoated HBC scatterer (*σ*_UC_) as calculated with the full scattering theory for a multilayer elastic cylinder^24^,^25^,^26^.


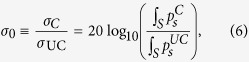


where 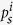 is the time averaged, scattered pressure amplitude, *i* = [*C*,*UC*] respectively enumerate coated/uncoated objects, and the contour *S* is a closed circular path surrounding the scatterer. Near the center of Fig. 2a, the non-resonant cancellation region is observed, which is well described by Eqs. (2)–(4), 7, 10 for both the quasi-static (“o”) and thin shell (“x”) expressions due to our low 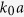 and moderate coating thickness. Selecting coating materials with physical properties close to those given by Eqs. (2)–(4), 7, 10 is an important part of ensuring broadband, non-resonant cancellation. An arbitrary search of the parameter space can lead to local minima in the scattering strength, which give inherently narrow band scattering reduction (or even enhancement) due to modal anti-resonances^19^. These regions are labeled in Fig. 2a and are far away from our region of interest. From this analysis it can be observed that simultaneous monopole and dipole cancellation in water can be achieved using a neutrally buoyant hollow shell and a coating material with a bulk modulus below, and a density near, that of water.

### Modeling a realistic coating

Compliant silicone rubbers can have a static density near water, high compressibility compared to buoyant metal shells and water, as well as a low shear modulus. In our frequency range of interest, silicone based rubbers also have a small, but non-zero, shear loss tangent which helps in damping the residual shear mode scattering. Collectively, these properties make them intriguing candidates for thin monopole cancellation coatings on metal HBC shells. In Fig. 2b we show the effective material properties for a silicone rubber coated, neutrally buoyant, stainless steel HBC (*ϕ*_*s*_ = 0.88) as a function of coating thickness. These effective values were extracted from the analytic scattering theory, with no material loss. The cancellation condition is met in Fig. 2b when 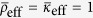. Thus, with thin shells of a commercially available silicone rubber (material properties listed in methods section) the appropriate condition for cancellation from a neutrally buoyant stainless steel shell is found to be a coating of thickness ratio *T*/*a* = 0.25.

The commercial finite element method (FEM) solver COMSOL was used to explore the effects of various coating thickness on cross-section reduction for specific sample sizes. Two-dimensional (2D) FEM simulations were used to include material losses in fully elastic material components, and to model the scattering from arrays of multiple HBC scatterers. This allowed a direct comparison to our experimental results. In Fig. 2c, we plot the scattering reduction, *σ*_0_, for a coated, neutrally buoyant, steel shell (outer radius *a* = 4 mm) as a function of coating thickness, T, at four frequencies 10 kHz, 20 kHz, 30 kHz, and 40 kHz. Care was taken to select frequencies for which there were no intrinsic resonances in the uncoated HBC, and the contour *S* is a closed circular path with radius *R* = 150 mm. As plotted in Fig. 2c, strong (40 dB) reduction of the total scattering cross-section is seen at 10 kHz (*k*_0_*a* = 0.2) and continues for all frequencies below 10 kHz. This broadband reduction in scattering falls-off as we move up in frequency, but still remains significant at 40 kHz (*k*_0_*a* = 0.85) where the optimal coating thickness produces an 18 dB reduction in the scattered field amplitude. At frequencies larger than 40 kHz higher order modal contributions reduce the significance of the monopole cancellation.

This is further elucidated in Fig. 3a,b in which scattered pressure amplitude color maps are plotted for the silicone coated HBCs at 10 kHz, 20 kHz, and for an uncoated HBC at 20 kHz in Fig. 3c. The cylinders are in water and the excitation source is a plane wave incident from the left with the amplitude normalized to the incident plane wave. The spatial distribution of the pressure amplitude strongly correlates with the far-field scattering pattern plotted in Fig. 3d. In Fig. 3d the angular distribution of the far-field sound pressure level is plotted in dB for coated and uncoated HBC at 10 kHz and 20 kHz. The uncoated HBC shows a uniform (monopole) scattering, while the coated HBC at 10 kHz has a residual, quadrupole-like pattern and the coated HBC at 20 kHz has a strong dipole contribution. At 10 kHz the far-field scattering pattern is modified from a pure quadruple pattern by interaction with a weak residual dipole. The increased strength of the 20 kHz dipole scattering, while still 30 dB less than the uncoated case, is due to the increasing mismatch between the dynamic and static density of the coated HBC and the aqueous background medium.

## Experimental results

**Figure 1:**
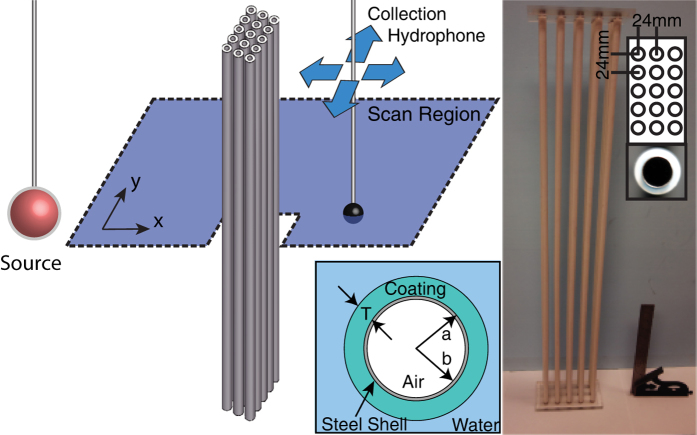
Experimental setup, geometry of coated shells, and image of the silicone coated array. The spherical source is located 1.1 m from sample along the x-axis, and the collection plane is vertically centered on sample. A 30 cm ruler is included for scale. The array geometry, and a single, coated, 8 mm outer diameter, stainless steel shell image is inset.

The scattered pressure field of objects with *k*_0_*a* << 1 can be small. This makes measurement a challenge in laboratory experiments. Edge effects from the finite length cylinders used in experiments also introduce additional scattering into any collected data. To overcome environmental noise and minimize these aperture effects, we increase the total scattered field of the HBC by using multiple coated and uncoated HBCs arranged into 5 × 3 rectangular arrays (as illustrated in Fig. 1). Sets of 5 × 3 arrays (with a silicone coating and without) were prepared for direct comparison between coated and uncoated scattering cases. The arrays were sequentially submersed in our 6 m × 6 m × 4 m water tank facility, and pressure amplitude maps were collected at the cylinder midpoint plane using a computer controlled, motorized hydrophone as shown in Fig. 1. Two silicone coating thicknesses were used to test the analytical results. A *T* = 0.8 mm and a *T* = 1.5 mm coating were assembled as described in the methods sections. A spherical source was used to generate an incident tone pulse from 10 kHz–40 kHz. The total pressure amplitude fields for the uncoated and *T* = 0.8 mm coated 5 × 3 cylinder arrays are plotted in Fig. 4, along with corresponding 2D FEM simulated total pressure fields. We used a coating thickness of *T* = 0.8 mm in the FEM simulations to match the stretched thickness of the silicone rubber shells.

**Figure 2:**
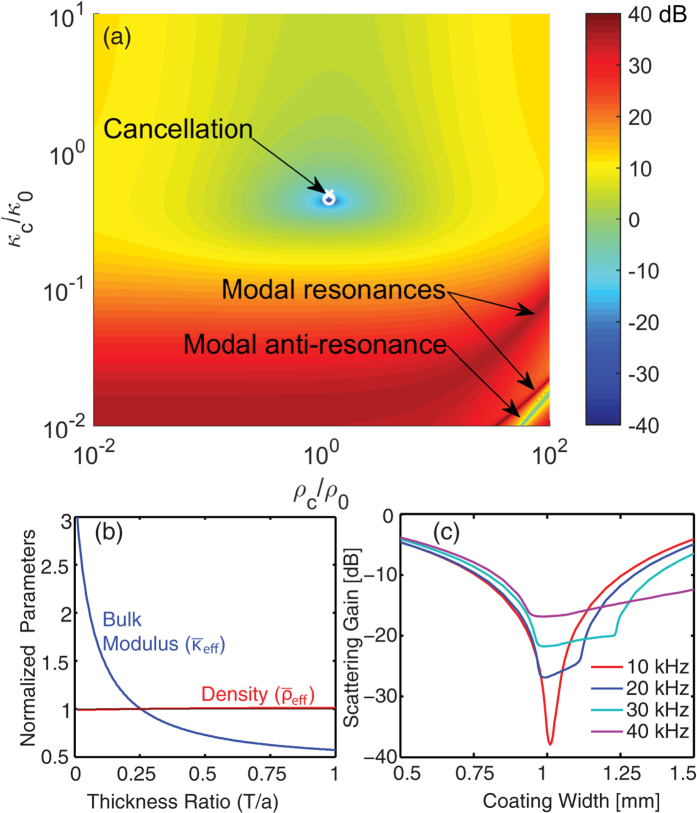
(**a**) Analytic calculation of scattering cross-section reduction of a coated neutrally buoyant stainless steel shell as a function of scaled coating parameters at fixed 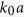 and *ϕ*_*c*_. Non-resonant cancellation and resonant modal cancellation/enhancement regions are indicated. (**b**) Extracted effective bulk modulus and effective density for lossless silicone rubber coating, on a neutrally buoyant HBC, as a function of thickness, T. (**c**) 2D FEM calculated scattering cancellation *σ*_0_, of a neutrally buoyant, stainless steel HBC, as a function of silicone coating thickness for frequencies 10 kHz–40 kHz.

**Figure 3:**
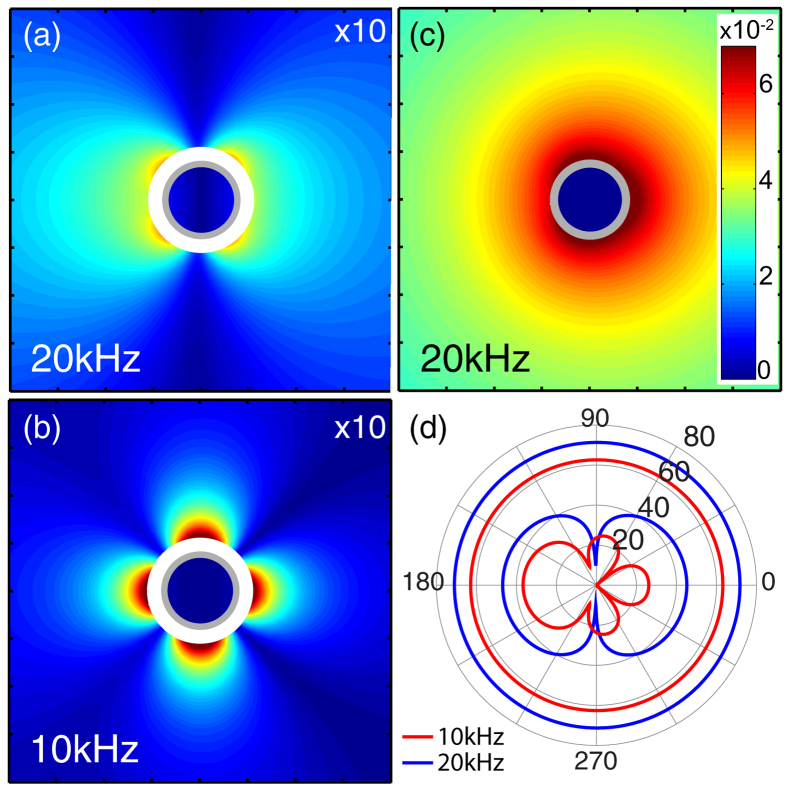
(**a**) 2D FEM calculated, scattered near-field pressure amplitude from silicone coated HBC with optimized thicknesses at 20 kHz (T = 1.035 mm) and (**b**) 10 kHz (T = 1.065 mm). (**a–c**) are plotted with the same color map with (**a**,**b**) scaled up by 10. (**c**) Scattered near-field pressure amplitude from uncoated HBC at 20 kHz and (**d**) angle resolved far-field sound pressure level (dB) of coated and uncoated HBC at 10 kHz (red) and 20 kHz (blue). Sound pressure level values are referenced to 1 *μ*Pa. In all cases, the outer diameters (2*a*) of the plotted cylinders are 8 mm.

**Figure 4:**
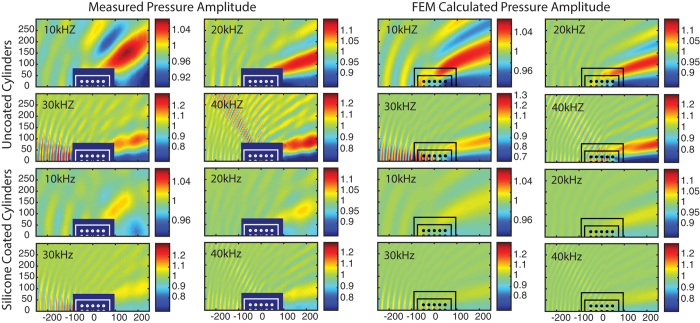
2D FEM calculated (right column) and experimentally measured (left column) pressure amplitude maps of uncoated and coated HBC arrays, with pressure amplitude indicated by the color bars, and incident frequency as indicated. The sample size and position are overlaid in each graph. The dark blue rectangle is the zone excluded in the experimental scan.

**Figure 5:**
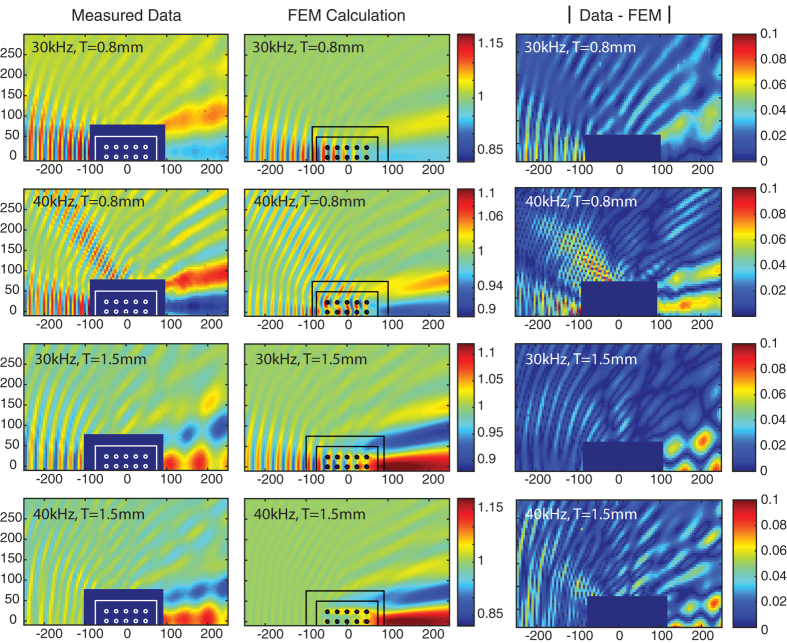
Measured and FEM calculated total pressure amplitude with a 5 × 3 array of silicone coated HBC with *T* = 0.8 mm, and *T* = 1.5 mm at 30 kHz and 40 kHz. The measured data (left column) and FEM results (middle column) are plotted on the same color scale for each frequency. The absolute value of the difference between the data and FEM plots are shown on in the right-most column.

In Fig. 5 we have plotted total pressure field collected from the *T* = 1.5 mm and *T* = 0.8 mm coated, 5 × 3 arrays of HBC at 30 and 40 kHz, and the corresponding 2D FEM simulation results. The salient features of this data are the continued agreement with the FEM simulations, and the existence of the forward scattering peak for *T* = 1.5 mm. In Fig. 5 the *T* = 0.8 mm data has been replotted from Fig. 4 with the range of the color map reduced to highlight the effects of cylinder array residual scattering. The forward scattering peak seen in the *T* = 1.5 mm data replaces the forward scattering shadow seen in the *T* = 0.8 mm and uncoated data results. As shown in Fig. 2b, because the *T* = 1.5 mm coating is larger than the optimal cancellation thickness, its effective bulk modulus is lower than the background bulk modulus. Conversely, for the *T* = 0.8 mm sample, Fig. 2b indicates an effective bulk modulus larger than the water background. For both types of cylinder, the effective density remains roughly equal to the background water medium, and therefore the effective sound speeds change from a speed less than water for the *T* = 1.5 mm sample array, to an effective sound speed above water within in the *T* = 0.8 mm sample array. This relative sound speed contrast causes the wave to advance within the array effective medium in the first case and retard in the second. We thus see a phase change in the scattered wave as the coating thickness changes from one side of the ideal geometry for cancellation to the other in Fig. 2c. The forward scattering peak seen in Fig. 5, in conjunction with the scattering shadow, confirms the existence of an ideal cancellation thickness between the tested *T* = 0.8 mm and *T* = 1.5 mm thicknesses.

### Comparing Experiment and Simulation

The results in Figs 4 and 5 show that there is good agreement between the measured and 2D FEM computed total pressure fields. The additional modulation of the total pressure amplitude in the collected data is due to the aperture diffraction from the finite length of the scattering cylinders and the additional scattering from the acrylic end caps. This can be seen in the difference plots of Fig. 5. These plots show the absolute value of the measured pressure amplitudes minus the FEM calculated quantity. Just as in Fig. 4 above, the physical parameters used are the values listed in the below methods section. In these difference plots one can see that the most significant deviations from the calculated FEM data are the modulated forward scattering lobes, which are caused by the aperture effects of our finite length samples^27^. As pure 2D simulations the FEM pressure fields do not include this modulation. Calculation of the standard deviation indicates that *σ* < 0.017 for all four difference plots shown in Fig. 5. As can be seen from the color map ranges, the maxima of the modulation due to the effects of the finite samples are at most 10% of the amplitude of the incident pressure. Although this value is not negligible, modulations of similar amplitude can be observed when comparing the experimental and simulated scattering lobes of the uncoated HBCs in the top panels of Fig. 4. We emphasize that the modulation of the forward scattering lobes imposed by the aperture diffraction is expected even for the case of impedance-matched apertures^27^. The forward-restricted diffractive modulations are also not of sufficient magnitude to mask the significant scattering reduction clearly observed at all angles in Fig. 4.

**Figure 6:**
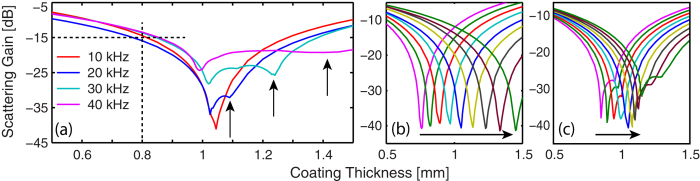
(**a**) FEM calculated scattering gain from an array of silicone coated HBC. (**b,c**) Effect of scaling coating physical parameters in (**a**) from 90% to 110% of original value at 10 kHz in 2.5% increments for (**b**) compression sound speed and (**c**) density. Arrows indicate (**a**) positions of enhanced backscattering reduction (**b,c**) direction of increased scaling.

Stretching of the silicone coating onto the HBC may have further impacted the experimental results as straining the rubber likely modified the silicone mechanical properties. In Fig. 6 we plot the FEM simulated scattering cross-section ratio, *σ*_0_, for 5 × 3 arrays of cylinders as a function of coating thickness. In Fig. 6a, calculation of the scattering cross-sections from the FEM simulation results indicate that the change between the uncoated and coated arrays for this non-optimal coating thickness of *T* = 0.8 mm is still ≈ −15 dB over the 10 kHz to 40 kHz range tested. Arranging the cylinders into an array with 24 mm spacing does not degrade the results of monopole cancellation. To account for possible variation in the coating physical parameters, we have also independently simulated the variation of the silicone sound speed and density, and plotted the resulting scattering reductions in Fig. 6b,c. The plots of Fig. 6b verify that an increase in compressional sound speed will degrade the cancellation efficiency at the fixed coating thickness of 0.8 mm. An increase in the compression modulus is the most likely result of the strain induced by stretching the silicone onto the HBC. This may be a contributor to the increased experimental scattering shown in Fig. 4 as compared to the calculated results, and the decrease in experimental forward scattering seen in Fig. 5. Due to the extended size of the array, at frequencies above 10 kHz the change in scattering cross-section is a more complicated function of coating thickness than for the single cylinder case. The addition of a second reduction peak in the total scattering cross-section gain at higher coating thicknesses in Fig. 6a is due to the enhanced cancellation of the back-scattered acoustic wave at the expense of forward-scattering reduction. This effect can be seen in the enhanced backscattering reduction between the 40 kHz and 30 kHz data plotted in Figs 4 and 5.

## Discussion

Although our experimental results were not conducted with the ideal coating thickness, the high degree of correlation between the observed and calculated pressure fields indicates that our FEM results are valid. We conclude that for a highly optimized shell thickness of 1 mm ± 40 *μ*m the complete monopole cancellation would result in a strong (>20 dB) reduction for all frequencies less than 40 kHz. In addition, the use of the double-shell arrays presented here can be utilized to engineer acoustic metafluid lattices with a gradient refractive index that simultaneously preserve buoyancy. As is evident in Fig. 2b, the effective bulk modulus of a coated HBC can be tuned by changing a single parameter (coating thickness) without changing the density-matched condition. Therefore, the local effective sound speed can be tailored throughout a sonic crystal lattice of coated HBC scatterers by simply changing the coating thickness of each scatterer. For example, it may be possible to make a buoyant version of the coordinate transformation which maps cylindrical waves to plane waves^28^,^29^ using a coated HBC lattice. Given that many aqueous systems are designed to preserve a static position underwater, the preservation of near-buoyancy should be of practical importance.

In conclusion, our computational and experimental results demonstrate that acoustic monopole scattering from hollow, cylindrical, metal cylinders can be canceled in an aqueous environment by the addition of a properly tailored compliant silicone elastomer shell. This coating scales the system’s effective bulk modulus while minimally impacting the total effective density. By starting with the condition that the hollow cylinder’s effective density be matched to the background via tailoring its geometry^18^, we have independently eliminated the dipole scattering mode. With this approach we have simultaneously matched the coated HBC’s acoustic impedance and sound speed to the background medium. Our computational results indicate that the scattering reductions possible are greater than 20 dB for arrays of optimally coated hollow cylinders up to *k*_0_*a* = 0.85 and scattering reductions greater than 40 dB are possible for coated HBCs with *k*_0_*a* ≤ 0.2. Our experiments have shown robust scattering reductions which occur even for coating thicknesses that are not fully optimized, and we have demonstrated a coating with a far-field scattering cross-section reduction of 15 dB.

## Methods

### Assembly of cylinder arrays

Buoyant 304 stainless steel tubes (ID 3.74 mm/OD 4 mm) were cut into 900 mm lengths to approximate the simulated 2D HBC system. We used commercially available silicone rubber tubing (McMaster Carr 5054K814) with ID 7 mm/OD 9 mm and an A35 Shore hardness rating as the cancellation shell. After initial stretching of 0.5in lengths onto the steel cylinders, the silicone tubing, inflated with 10 psi compressed air and lubricated with isopropanol, was pushed onto the end-capped steel cylinders. The size mismatch of the silicone tubing ID/steel cylinder OD ensured no air gaps in the resulting coating, however the silicone wall thickness was stretched to *T* = 0.8mm ± 50 *μ*m. We arranged the 900 mm long, hollow, stainless steel cylinders in a rectangular lattice of 6 mm high posts machined into the surface of a 12 mm thick acrylic sheet. These posts provided the repeatable, fixed geometry locations for the arrayed hollow steel cylinders. The cylinders were spaced 24 mm apart to minimize any near-field coupling between the individual cylinders. Polydimethylsiloxane (PDMS) was then poured into the acrylic end blocks to seal the cylinders against water intrusion.

### Measurement of acoustic scattering

We vertically centered a 100 mm diameter spherical source 1.1 m from the 5 × 3 array of (un)coated cylinders. A computer controlled, Velmex VXM 3-axis motion stage was used to position a B&K 8103 hydrophone at the vertical center of the cylinder array. The hydrophone was scanned in the horizontal plane perpendicular to the cylinder array long axis and the collected pressure data was amplified and digitized at a rate of 10^6^ samples/second. The monopole source was driven with a 30 cycle, cosine tapered, tone pulse at 10 kHz, 20 kHz, 30 kHz and 40 kHz. The time averaged pressure amplitude was extracted from the data during the window of incident/scattered wave temporal overlap and corrected for the 1/*R*^2^ intensity fall off of the spherical source. Additionally, pulse-to-pulse variation in the source output amplitude was corrected for with a fixed position hydrophone.

### Finite element calculations and material properties

In both analytic and FEM calculations, air-filled steel cylinders were simulated with a steel density equal to 7850 kg*/*m^3^, a Young’s modulus of 210 GPa, and a Poisson’s ratio of 0.3. The silicone rubber properties used were a density of 1020 kg*/*m^3^ and a compressional sound speed of 980 m/s^30^. Neither loss parameters nor shear modulus were available for the silicone rubber used. However, a shear-wave speed of 30 m/s and a shear loss tangent of 0.15 were estimated for FEM calculation based on measured values for a similar silicone rubber (polydimethylsiloxane)^31^,^32^. The background medium is water at standard temperature and pressure. A background plane wave was incident on the scatterer, and we solved for the scattered pressure to minimize computational error of the weakly scattered field.

## Additional Information

**How to cite this article**: Rohde, C. A. *et al.* Experimental Demonstration of Underwater Acoustic Scattering Cancellation. *Sci. Rep.* **5**, 13175; doi: 10.1038/srep13175 (2015).
